# Age-related Changes in Ongoing Thought Relate to External Context and Individual Cognition

**DOI:** 10.1093/geroni/igab046.3460

**Published:** 2021-12-17

**Authors:** Adam Turnbull, Giulia Poerio, Feng Lin, Jonathan Smallwood

**Affiliations:** 1 University of Rochester Medical School, Rochester, New York, United States; 2 University of Essex, Colchester, England, United Kingdom; 3 Stanford, Stanford, California, United States; 4 Queen's Universtiy, Kingston, Ontario, Canada

## Abstract

Understanding how age-related changes in cognition manifest in the real world is an important goal for aging research. One means of capturing these changes involves “experience sampling” participant’s self-reported thoughts as they go about their daily lives. Previous research using this method has shown age-related changes in ongoing thought: e.g., older adults have fewer thoughts unrelated to the here-and-now. However, it is currently unclear how these changes reflect cognitive aging or lifestyle changes. 78 younger adults and 35 older adults rated their thought contents along 20 dimensions and the difficulty of their current activity in their daily lives. They also performed cognitive tasks in the laboratory. In a set of exploratory analyses using Principal Component Analysis (PCA), we found that older adults spent more time thinking positive, wanted thoughts, particularly in demanding contexts, suggesting they may use different strategies to regulate their emotions. In line with previous research, older adults spent less time mind wandering about their future selves. Past-related thought related to episodic memory differently in older and younger adults. Additionally, PCA analyses performed separately in older and younger adults showed high similarity to an analysis performed on the combined sample, suggesting a similar structure to ongoing daily life thought in older and younger adults. These findings inform the use of experience sampling to understand cognitive aging, highlighting the need to consider content along multiple dimensions as well as the context in which thoughts are reported when analyzing aging ongoing thought.

